# Effect of the sanitation, hygiene, information, and education intervention on WaSH practices and related health outcomes among children in rural Anganwadi centres: a non-randomised cluster trial pilot tested in Odisha, India

**DOI:** 10.3389/fpubh.2025.1676981

**Published:** 2025-12-01

**Authors:** Sonali Kar, Snigdha Singh, Angana Ray, Baishnabi Pattnaik, Sumelika Das, Ayesha Das, Rosy Nayak

**Affiliations:** Department of Community Medicine, KIMS, KIIT University, Bhubaneswar, Odisha, India

**Keywords:** WaSH practices, AWC, diarrhoea, ARI, sanitation, hygiene, information, education

## Abstract

**Background:**

Inadequate water, sanitation, and hygiene (WaSH) facilities in Anganwadi centres, critical components of India’s Integrated Child Development Services (ICDS), adversely affect child health. The SHINE (Sanitation, Hygiene, Information, and Education) intervention aimed to improve WaSH practices and related health outcomes in rural Anganwadis of Odisha.

**Methods:**

This was a quasi-experimental, non-randomised cluster study conducted between April and October 2024 across four Anganwadi centres under the Rural Health Training Centre (RHTC), Kalinga Institute of Medical Sciences (KIMS), Odisha. Clusters were defined geographically: centres within 5 km of RHTC were assigned to the intervention arm, while those beyond 5 km served as controls. This distance criterion was selected to reduce the contamination risk of the intervention. Due to the nature of the intervention, blinding was not feasible.

**Results:**

Forty-five children were enrolled (intervention: 23 and control: 22). The intervention group showed significant improvements in safe drinking water usage (from 0 to 60.9%, AOR = 6.88), footwear use in toilets (17.4 to 78.3%, AOR = 5.18), and handwashing before meals (0 to 82.6%, AOR = 6.85). Infection symptoms declined markedly (from 39.1 to 4.4%, AOR = 5.18). Improvements in food hygiene and school infrastructure were observed. Absenteeism decreased, but not significantly. Control schools showed modest improvements, possibly due to the Hawthorne effect.

**Conclusion:**

The SHINE intervention significantly improved WaSH practices and reduced illness among Anganwadi children. The findings support integrating behaviour-centred WaSH interventions into early childhood education programs to promote health and reduce preventable disease in low-resource settings.

## Introduction

Anganwadis are a part of the Integrated Child Development Services (ICDS) program in India that play an important role in the early life development of a child by providing education, nutrition, and healthcare services. However, many of these centres lack adequate water, sanitation, and hygiene (WaSH) infrastructure, compromising their effectiveness. WaSH interventions are crucial in improving health outcomes, particularly among children in resource-poor settings. A deficit in WaSH facilities significantly leads to the global burden of disease, with the under-5 being disproportionately affected (1). It accounts for one of the highest childhood mortality from respiratory and gastrointestinal causes globally and in India; the situation, though controlled, demands improvement (2–4). Recognising this, the government has initiated efforts to establish standardised guidelines for constructing and maintaining water sources, toilets, handwashing stations, and hygiene promotion programs in Anganwadi centres.

A gap is perceived in the implementation and evaluation of such interventions at the Anganwadi level in rural India. Globally, there is mixed evidence on the impact of WaSH on school health outcomes; some studies report significant improvements in the health and school attendance of children, while other research shows no impact (5, 6). By assessing the impact of the intervention on students’ WaSH practices, health outcomes, and school attendance, the study seeks to provide evidence-based recommendations for scaling up similar interventions in other rural settings. Instilling proper hygiene practices from an early age can lead to long-term health benefits, thereby contributing to breaking the cycle of poverty and disease (7).

This study aimed to evaluate the effectiveness of local WaSH-related interventions at Anganwadi schools in rural settings. The intervention included water, hygiene, and sanitation infrastructure, as well as education offered as part of the Art of Giving campaign of the Mother Institute. This initiative was structured to promote behaviour change among students from an early phase of life and to encourage its acceptance by the primary caregivers of the participating students at home. The study hypothesis was that the WaSH intervention: Sanitation, Hygiene, Information, and Education-“SHINE” would improve the practices under study among the schools and students and also have an impact on their school attendance and overall health.

## Methods

### Study design and setting

This non-randomised cluster-controlled study was conducted from April to October 2024 in the catchment area of the Rural Health Training Centre (RHTC), Kalinga Institute of Medical Sciences (KIMS), Odisha. Of 10 Anganwadi schools in the catchment area of RHTC, 7 were eligible for the study as the other 2 Anganwadi’s were facing administrative issues, and 1 was undergoing renovation during the study recruitment period. Four Anganwadi centres were randomly selected from 10 eligible centres. Centres within 5 km were allocated to the intervention arm, while those beyond 5 km served as controls, minimising contamination risk. The study was done as a part of an Art of Giving Initiative, which was a novel endeavour of the University to offer its empathy and concern to the underserved.

### Participants and randomisation

All students enrolled in the selected Anganwadi centres were included (*n* = 45). The intervention group (*n* = 23) and the control group (*n* = 22) were balanced for age and gender. School-level consent (from parents) was obtained from the principals.

### Intervention—the SHINE package

The intervention package “SHINE pack” (Sanitation, Hygiene, Information, and Education) comprised a combination of behaviour change promotion activity and provision of logistics such that drinking safe water, maintaining sanitation, and hygiene become normal behaviour. The elements of the package were as follows:

Safe drinking water—A water filter was provided by the team, placed at an easily accessible space for the children and away from potential sources of contamination/pollution, in addition to providing education regarding safe drinking water practices to the children and the workers at the Anganwadi.
Toilet sanitation and hygiene—Toilet cleaner and brush, and rubber footwear for the children were provided along with an awareness session on toilet sanitation and hygiene, and the importance of using the footwear while going to the toilet.
Hand washing facility—Handwash liquid and soaps, and posters illustrating the steps of handwashing were provided for the children to wash their hands before and after having food, and after using the toilet. An education session regarding the importance of the same was given to the children and the workers.
Food hygiene—Provision of plates and glasses for every student, along with a disposal bin, was made available at the dining area of the Anganwadi. Education was given regarding eating in an area that is clean and free from contamination, use of cleaned plates and drinking glass, keeping food covered while storing, and disposal of food waste in bins only.

The orientation and resource content were developed, ensuring technical guidance from the manuals issued by the Ministry of Health & Family Welfare (MoHFW), India, and were completely theme-based, need-specific, and customised, considering the age of students at Anganwadi. The design was formed referring to the behaviour-centred design approach that guided behaviour alteration via environmental or facility cues, along with activity-based curriculum, and motivators. The elements of the government-run program through the Sarva Shikshya Abhiyan already had mandates of the availability of toilets, running tap water supply in the schools, whereas the control schools had only these mandates.

All eligible Anganwadis under the catchment of RHTC had consented to receive the SHINE Package. After the random allocation was conducted, formal consent for participation was sought from school principals in the form of *loco parentis*, on behalf of all students in their school, and parental information sheets regarding the study were collected by parents from the principal.

Using a semi-structured, mixed checklist in the first visit to all four Anganwadis, (a) WaSH practices and baseline characteristics of students at Anganwadi, and (b) WaSH facilities at Anganwadi, were assessed. If a student was absent from school on this day, he/she was to be interviewed at home. However, such a situation did not arise. The teachers and other staff, including the food handlers in all these schools, then received training focused on safe water, food hygiene, handwashing, personal hygiene, and toilet hygiene and sanitation, irrespective of intervention allocation. The socio-demographic information of the student, household WaSH access and practices, number of full/partial days absent in the last 7 days, reason for absence, and symptoms of any infectious disease over the last 7 days were reported by the parents. The study team conducted home visits with parents, which lasted between 25 and 40 min each.

Following the baseline assessment in the first month, the intervention arm received the information and education component of the SHINE package monthly over a period of 3 months, while, except for the handwashing soap/liquid, the logistics—IEC materials, water filter, food plate and glasses, and bin—were given only once after the baseline assessment in the first visit. The impact of the intervention, in addition to routine government-run tools for hygiene and sanitation, was assessed through observation during three unannounced follow-up visits to the school using a checklist. The unannounced check on attendance was applied to avoid bias often associated with absence measurement.

Behaviour-centred design principles guided content delivery. The intervention included monthly behaviour change sessions and one-time distribution of logistical items. The above assembled package was offered to the Interventional AWC during monthly visits by a supervisory team comprising the authors and interns. The control AWCs were also visited monthly and offered the routine health checkups and demand-driven counselling.

As part of an awareness exercise during the 2024 World Health Day observation, this study was conducted after obtaining permission from the Institutional head and department head. As it was an academic exercise, ethics approval was waived. Written informed consent was obtained from the principals of the participating schools. Confidentiality was maintained for the schools’ and participants’ information.

### Data collection and analysis

Data collected were entered into a Microsoft Excel sheet and checked for missingness, data distribution, and outliers. The completers-only framework was used to analyse the data using STATA 17. In the current study, no participants dropped out. Analysis was conducted to assess the effect of the intervention by study arm over time, specifically during the first and third month visits. Multivariate logistic regression was performed for all outcomes, except for absence from the Anganwadi school in the last 7 days, for which linear regression was used. The effect of the intervention on the primary outcomes was calculated using a model that adjusted for the age and socio-economic status of the participants. For the secondary outcomes, the model was adjusted for school size.

## Results

**Figure 1:**
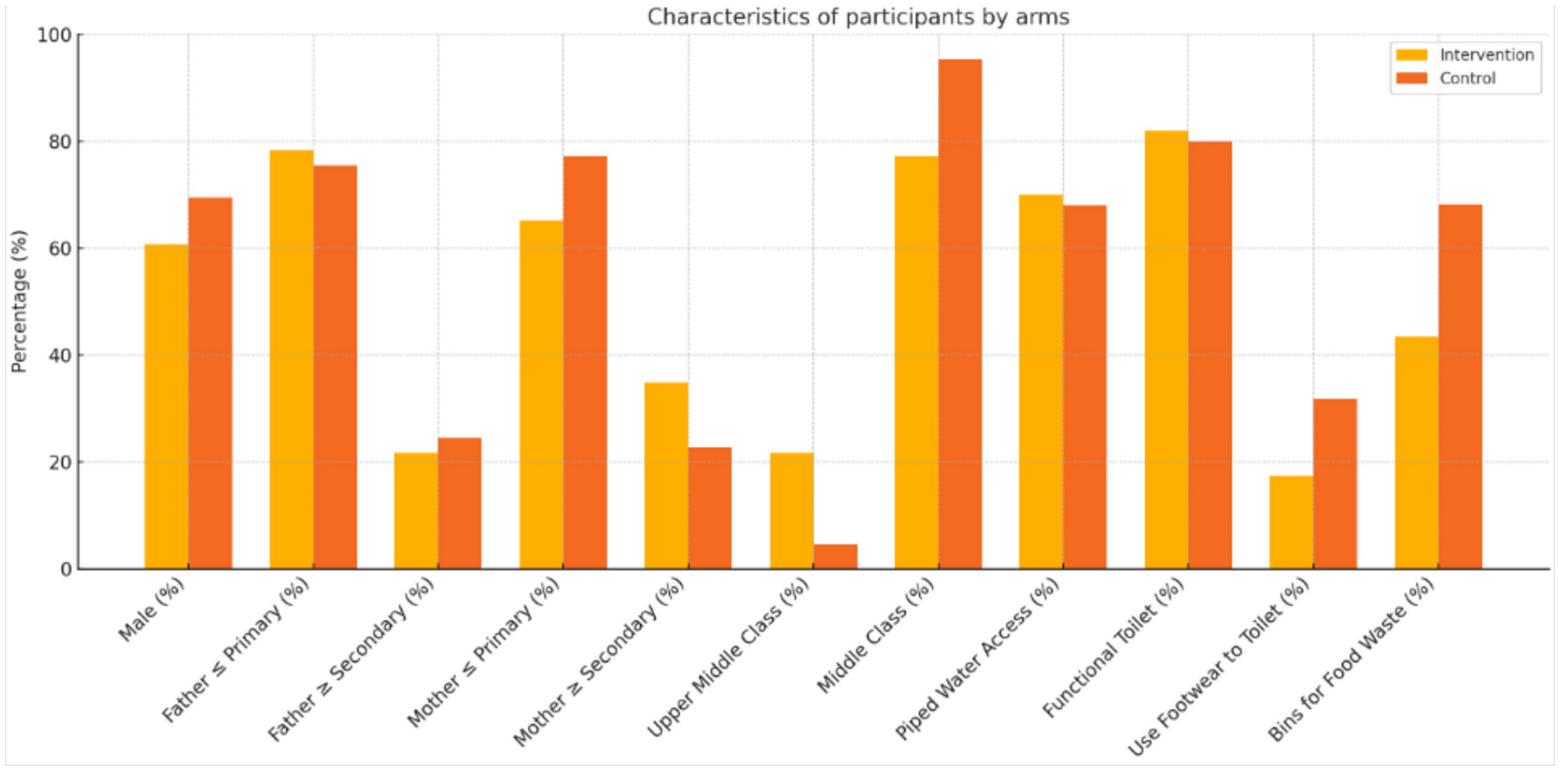
Characteristics of participants by arms.

None of the four centres that were randomly selected and consented to participate withdrew from the study. A total of 45 students were enrolled in this study. Twenty-three students received the intervention, while 22 did not, as they were from the two Anganwadis in the control arm (Figure 1).

Table 1 depicts the characteristics of study participants in the intervention and control arms. The mean age of participants in the intervention group was found to be higher (4.8 ± 1.2 years) compared to the control group (3.9 ± 1.3 years). The proportion of male participants in the two groups (60.8% vs. 69.5%) and parental education were similar, with a majority having completed primary education. Upon analysing the socioeconomic status, 21.7% of participants belonged to the upper middle class in the intervention arm compared to 4.6% in the control arm, which is statistically significant (*p* = 0.01). Access to WaSH-associated household infrastructure was also uniformly distributed across the two arms. However, in the control arm, a slightly higher proportion of participants reported using footwear while going to the toilet (31.8% vs. 17.4%) and having bins for food waste disposal at their home(68.2% vs. 43.5%).

**Table 1:** Characteristics of participants by arms.

Variables	Intervention arm (*n* = 23)	Control arm (*n* = 22)	*p*-value
Mean age (years)	4.8 ± 1.2	3.9 ± 1.3	0.03
Male	14 (60.8%)	15 (69.5%)	0.76
Father’s education^a^			
≤ Primary	18 (78.3%)	17 (75.5%)	0.11
≥ Secondary	5 (21.7%)	5 (24.5%)	
Mother’s education^a^			
≤ Primary	15 (65.2%)	17 (77.3%)	0.23
≥ Secondary	8 (34.8%)	5 (22.7%)	
Socio-economic status^b^			
Upper middle class	5 (21.7%)	1 (4.6%)	0.01
Middle class	17 (77.3%)	21 (95.4%)	
Household with piped water access	16 (70.0%)	15 (68.0%)	0.88
Household with a functional toilet	19 (82.0%)	18 (80.0%)	0.54
Use of footwear while going to the toilet	4 (17.4%)	7 (31.8%)	0.06
Household with bins for food waste disposal	10 (43.5%)	15 (68.2%)	0.05

a Indian standard classification of education.

b Modified BG Prasad scale (2021).

Tables 2, 3 are dynamic and capture data across three observation points (1 month apart) post-intervention in the intervention group. They demonstrate the magnitude of intervention effects compared to controls, adjusting for baseline differences where applicable. Age of the participant was *a priori* while building the regression model. Table 1 suggests that those in the intervention group were relatively more affluent than those in the control group in terms of the Socio-economic status (SES) of the participants, which might have an impact on hygiene practices and access to infrastructure. Thus, the socioeconomic status of participants was considered a potential confounder in the analysis of the intervention effect.

**Table 2:** Effect of intervention on WaSH practices over time among the participants^a^ (post-intervention).

Outcome measure	At baseline	Time point 1 (4th months)	Time point 2 (5th months)	Time point 3 (6th months)	Adjusted odds ratio (95% CI)^b^	*p* value
Safe drinking water						
Drinking water from water filters						
Intervention arm	0 (0%)	5 (21.7%)	8 (34.7%)	14 (60.8%)	6.88 (2.09, 22.16)	0.04
Control arm	0 (0%)	0 (0%)	0 (0%)	3 (13.6%)	1	
Toilet sanitation						
Wearing footwear to the toilet						
Intervention arm	4 (17.4%)	6 (26. %)	11 (47.8%)	18 (78.3%)	5.18 (1.82, 14.72)	<0.01
Control arm	7 (31.8%)	7 (31.8%)	9 (40.9%)	9 (40.9%)	1	
Hand washing practice						
Washing hands before eating						
Intervention arm	0 (0%)	5 (21.7%)	16 (69.6%)	9 (82.6%)	0.85 (2.12, 22.12)	<0.01
Control arm	0 (0%)	7 (31.8%)	4 (18.0.2%)	10 (45.5%)	1	
Washing Hands after using toilet						
Intervention arm	0 (0%)	2 (8.7%)	8 (34.8%)	12 (52.2%)	3.32 (0.26, 6.59)	0.15
Control arm	0 (0%)	0 (0%)	2 (9.10%)	5 (22.7%)	1	
Food hygiene						
Eating in clean area						
Intervention arm	4 (17.4%)	6 (26.1%)	4 (17.4%)	10 (43.5%)	5.32 (1.68, 12.05)	0.43
Control arm	4 (18.2%)	2 (9.1%)	3 (13.6%)	5 (22.7%)	1	
Disposal of food waste in bins						
Intervention arm	0 (0%)	8 (34.8%)	10 (43.4%)	10 (43.5%)	7.52(2.98, 13.88)	0.01
Control arm	0 (0%)	0 (0%)	0(0%)	0 (0%)	1	
School attendance^c^						
Symptoms of infection in the last 7 days						
Intervention arm	9 (39.1%)	2 (8.7%)	3 (13. %)	1 (4.35%)	5.18 (1.82, 14.72)	0.04
Control arm	7 (31.8%)	4 (18.2%)	6 (27.3%)	4 (18.18%)	1	
Absence in the last 7 days (mean ± SD)					**Beta regression coeff. (95%CI)**	***p*-value**
Intervention arm	0.43 ± 0.11	0.50 ± 0.10	0.42 ± 0.10	0.35 ± 0.15	4.32 (0.05, 6.59)	0.23
Control arm	0.39 ± 0.15	0.32 ± 0.21	0.55 ± 0.45	0.43 ± 0.20		

a No. of participants in the intervention arm = 23 and in control arm = 22.

b Adjusted for age and socio-economic status.

c Adjusted for all other study outcomes.

**Table 3:** Characteristics of Anganwadi schools by study arm over time (post-intervention)^a^.

School WaSH indicator	At baseline	V1 (1 month)	V2 (2 months)	V3 (3 months)	Odds ratio (95% CI)^b^	*p* value
Functional handwashing stations (intervention schools)	0 (0%)	1 (50%)	1 (50%)	2 (100%)	9.12 (1.87, 44.42)	<0.01
Functional handwashing stations (control schools)	1 (50%)	1 (50%)	1 (50%)	1 (50%)	1	–
Provision of safe drinking water (intervention schools)	0 (0%)	1 (50%)	0 (0%)	1 (50%)	7.45 (1.95, 28.44)	<0.01
Provision of safe drinking water (control schools)	0 (0%)	0 (0%)	0 (0%)	1 (50%)	1	–
Availability of toilet cleaning supplies (intervention schools)	0 (0%)	1 (50%)	2 (100%)	2 (100%)	6.85 (2.12, 22.12)	<0.01
Availability of toilet cleaning supplies (control schools)	0 (0%)	0 (0%)	0 (0%)	1 (50%)	1	-
Teachers demonstrating hygiene practices (intervention arm)	0 (0%)	1 (50%)	2 (100%)	2 (100%)	9.12 (1.87, 44.42)	<0.01
Teachers demonstrating hygiene practices (control arm)	0 (0%)	0 (0%)	0 (0%)	1 (50%)	1	-

a No. of schools in the intervention arm = 2 and in the control arm = 2.

b Adjusted for school size.

Table 2 illustrates the effect of the SHINE package intervention on the WaSH practices of the participants over time, at baseline and during the 3 months post-intervention. AOR (adjusted odds ratios) reports the effect of intervention on the likelihood of students’ behaviour change over time, comparing the intervention arm to the controls.

Primary outcomes indicated from Table 2 and Figure 1:

- *Safe Drinking Water:* Intervention uptake increased to 60.9% versus 13.6% in control (aOR = 6.88; *p* = 0.04).
- *Toilet Sanitation (Footwear Use):* Rose to 78.3% versus 40.9% in control (AOR = 5.18; *p* < 0.01).
- *Handwashing Before Meals:* Increased to 82.6% versus 45.5% (AOR = 6.85; p < 0.01).
- *Infection Symptoms:* Dropped from 39.1 to 4.4% (AOR = 5.18; p = 0.04).
- *School Attendance:* Mean absence reduced (0.43 to 0.35 days), though not statistically significant (*p* = 0.23).

Thus, this study reports that the SHINE package significantly improved various hygiene practices and reduced symptoms of infection among children in the intervention group compared to the controls. Additionally, an improvement in school attendance was observed, although it was not statistically significant within this study period.

Table 3 shows a comparison of WaSH-related indicators in intervention and control arms at baseline and over a 3-month period following the intervention.

Intervention schools consistently outperformed control schools over time, showcasing the sustained impact of the SHINE package on WaSH-related practices and infrastructure.

**Figure 2:**
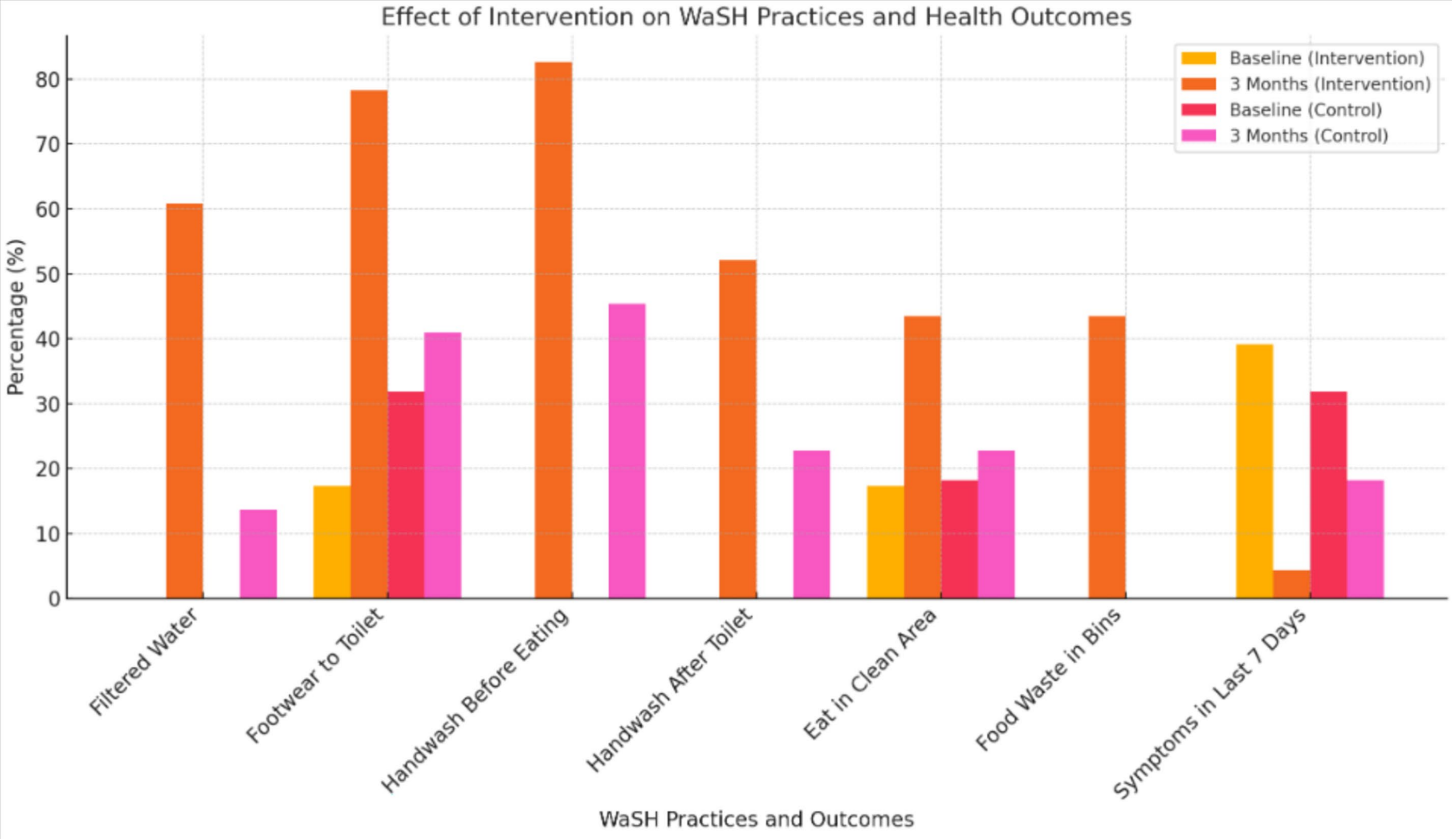
Effect of intervention on WaSH practices and health outcomes by study.

Secondary outcomes (school-level) as indicated in Table 2 and Figure 2:

- Functional handwashing stations, toilet cleaning supplies, and hygiene demonstrations increased significantly in intervention schools (all *p* < 0.01).
- Food hygiene improved (use of bins, clean eating areas), though with variable significance.

Control arm effects: despite no formal intervention, minor improvements were observed, suggesting an observer effect or indirect motivation.

## Discussion

This study demonstrated the feasibility and effectiveness of a comprehensive WaSH intervention (SHINE) in improving hygiene behaviours and reducing infection among preschool children in rural Odisha. Findings align with the global literature, which links hygiene interventions to a reduction in childhood illnesses, particularly gastrointestinal and respiratory infections (4–6).

The intervention’s strength lay in its holistic design—integrating infrastructure and behaviour change via tailored messaging and staff engagement (8, 9). Notably, indirect improvements in control centres suggest potential spillover effects or reactivity due to monitoring.

However, improvements in school attendance did not reach statistical significance, possibly due to the short follow-up duration or other unmeasured social determinants. Furthermore, although handwashing and safe water use improved significantly, limitations such as the lack of upgraded toilet infrastructure and the availability of these amenities at home may have constrained the impact on reducing diarrheal illness, as reported in other studies (10, 11). The major concern was the unstructured and disrupted supply of essential items to AWCs, as well as funding constraints for replacing these items, such as soap, cleaning materials, and repair work. The incentive-based payment of Anganwadi Worker (AWW) for various other maternal and child-based initiatives also perhaps deters them from focusing on AWC and the children coming there.

Interestingly, students from intervention schools mandated the WASH practices at their homes and among younger siblings which is similar to the findings in the study by Joshi and Amadi which found that school-based WASH interventions had a positive impact on hygiene behaviour outside of the classroom (12). Future interventions should consider scaling this integrated model while incorporating long-term monitoring and infrastructure development. Integration with national programs, such as ICDS, Sarva Shiksha Abhiyan, and RBSK (Rastriya Bal Suraksha Karyakram), can amplify reach and sustainability.

The SHINE package can be emphasised through Community-Based Organisations and medical colleges, where small investments can yield worthwhile benefits.

### Limitation

Due to the requirement for ethical permissions, the study was piloted in an Anganwadi Centre (AWC) attached to the Rural Health Training Centre (RHTC). The study period was short, and the intervention arm was a catchment area for the study institute; sustainability can be secured to some extent. However, in the control arms, introducing this package is more challenging. Thus, it has to be embedded in the Programme of Poshan 2.0, which calls for a Saksham Anganwadi.

## Data Availability

The raw data supporting the conclusions of this article will be made available by the authors, without undue reservation.
